# Case of Tetanus Successfully Treated by Sedative Remedies

**Published:** 1820-04

**Authors:** 

**Affiliations:** Fulignano.


FOR THE LONDON MEDICAL AND PHYSICAL JOURNAL.

*Case of Tetanus successfully treated by sedative Remedies.* By Dr. Cavallini, of Fulignano.

LEWIS FONZI, 50 years of age, of a vigorous temperament and athletic habit, wounded himself with a sharp instrument in the second phalanx of the third finger of the left hand. The wound was not deep, was not attended with any remarkable symptoms, and healed very well in the course of the morbid affection about to be described.

A few days after having received the wound he exposed himself to rain. When he went to bed in the evening, he was so wearied by fatigue, that, although the bed-coverings fell off in the course of the night, and he was exposed to extreme cold, he was not by this awoken, his sleep was so profound. He got up as usual in the morning, but felt himself very stiff and sore over the whole body. He exposed himself the two following days to the same circumstances as those above indicated; so that it became a matter of doubt, whether the tetanus that supervened should be attributed to these or to the wound.

After a few days the patient became affected with nausea, difficulty of respiration, and a sense of tension in the chest. A free blood-letting gave him decisive relief. On the evening of the third day from the bleeding, he was suddenly seized with violent pain in the region of the loins, principally on the left side, which, on the next day, became extended over the whole trunk, then to the neck, first producing trismus, and afterwards real emprosthotonic tetanus, attended with sense of great debility. A physician who was present at the onset of the disease, ordered immediately infusion of canella, opiates, frictions with camphorated alcohol, and the warm bath. The rigidity of the muscles increased under this treatment; physiological debility became augmented, and, on the third day, with the exception of the arms, the whole body was inflexible. The patient suffered very acute pains in the loins, extending to the scrobiculus cordis; and the skin was covered by a copious sweat. Dr. d'Ascoli and myself were now consulted. We agreed in opinion respecting the impropriety of stimulating treatment, and that sedative measures should be employed, which were immediately resorted to. This was the fourth day of the disease, when the pains were so violent, and the tetanic spasm so intense in degree, that the danger of death appeared to be imminent.

Thirty leeches were first applied to the chest, which prowe descend lower in the scale, we find animals capable of regenerating whole numbers; and at length some, the different parts of which, when they are cut into pieces, live as distinct animals.

duced some amelioration, and the patient was now able to drink a little water. A decoction of digitalis, with the addition of some cohobated laurel-water, were now administered, by which the relief obtained was confirmed. Encouraged by the favourable results of the contra-stimulant method, I determined to repeat the application of leeches in great numbers, first to the abdomen, and then along the vertebral column. One hundred and fifty were thus applied within a few days, and the blood permitted to flow freely from the punctures. A glyster composed of decoction of mallows, with half a drachm of tartar emetic, was injected twice a-day; and tartar emetic was also freely administered by the mouth. Mercurial frictions were made on the limbs, and the diet restrained to a little panada, with iced-water as drink. On the fifteenth day of the disease, (the eleventh after the use of the sedative treatment,) the diminution of the symptoms was very considerable, and the patient was evidently proceeding towards health. But the rigor of the diet being diminished, a little orgasm returned, which was removed by persisting with the antiphlogistic measures and cold aspersion. On the twenty-fifth day, the symptoms above described had disappeared, and the secretions and excretions in general had returned to the natural state. There remained, however, some morbid erethism in the gastro-enteric system, accompanied by development of gas, meteorism, and constipation of the bowels. Castor oil, manna, antimoniated tartrate of potash, cold fomentations, and purgative glysters, produced abundant evacuations, and completed the cure. Although the muscles generally had acquired their natural softness and flexibility, the skin was morbidly constricted ; but this soon disappeared : the muscles of the lower jaw were the last that remained contracted, but this finally recovered its natural power of motion. On the fortieth day, the patient left his bed, and began to assume his ordinary regimen.

